# Multi-Approaches Analysis Reveals Local Adaptation in the Emmer Wheat (*Triticum dicoccoides*) at Macro- but not Micro-Geographical Scale

**DOI:** 10.1371/journal.pone.0121153

**Published:** 2015-03-20

**Authors:** Sergei Volis, Danara Ormanbekova, Kanat Yermekbayev, Minshu Song, Irina Shulgina

**Affiliations:** 1 Key Laboratory for Plant Diversity and Biogeography of East Asia, Kunming Institute of Botany, Chinese Academy of Sciences, Kunming, 650204, China; 2 Institute of Plant Biology and Biotechnology, 45 Timiryazev St., Almaty 050040, Kazakhstan; University of Lausanne, SWITZERLAND

## Abstract

Detecting local adaptation and its spatial scale is one of the most important questions of evolutionary biology. However, recognition of the effect of local selection can be challenging when there is considerable environmental variation across the distance at the whole species range. We analyzed patterns of local adaptation in emmer wheat, *Triticum dicoccoides*, at two spatial scales, small (inter-population distance less than one km) and large (inter-population distance more than 50 km) using several approaches. Plants originating from four distinct habitats at two geographic scales (cold edge, arid edge and two topographically dissimilar core locations) were reciprocally transplanted and their success over time was measured as 1) lifetime fitness in a year of planting, and 2) population growth four years after planting. In addition, we analyzed molecular (SSR) and quantitative trait variation and calculated the *Q*_ST_/*F*_ST_ ratio. No home advantage was detected at the small spatial scale. At the large spatial scale, home advantage was detected for the core population and the cold edge population in the year of introduction via measuring life-time plant performance. However, superior performance of the arid edge population in its own environment was evident only after several generations via measuring experimental population growth rate through genotyping with SSRs allowing counting the number of plants and seeds per introduced genotype per site. These results highlight the importance of multi-generation surveys of population growth rate in local adaptation testing. Despite predominant self-fertilization of *T*. *dicoccoides* and the associated high degree of structuring of genetic variation, the results of the *Q*_ST_ - *F*_ST_ comparison were in general agreement with the pattern of local adaptation at the two spatial scales detected by reciprocal transplanting.

## Introduction

Plant species that are distributed over heterogeneous environments often show genetic/phenotypic differentiation resulting from either genetic drift or natural selection. Recognition and understanding adaptive differentiation (i.e. through diversifying selection) is an important and challenging task in evolutionary biology, because adaptation to the local environment is considered a major driving force of phenotypic change and speciation [1–5]. Local adaptation is by definition the outcome of an evolutionary process in which populations evolve towards a phenotype that has higher fitness in its local environment relative to the non-native environmental conditions, due to fitness trade-offs [6–9]. Local adaptation of plant populations has been well documented (reviewed in [4,10–11]) but the interaction of local selection with the geographic context is poorly understood [12]. Theory predicts that the magnitude of local adaptation increases with geographic distance separating populations, because of an increase in both environmental differences and genetic isolation [13–15]. However, there is little empirical support for this pattern (reviewed in [16]).

The lack of a relationship between geographic distance and probability of local adaptation is hypothesized to be either due to independence of the magnitude of environmental variation and geographic scale [15] or because of non-linearity of this relationship [17]. If environmental heterogeneity is high across a range of spatial scales within a species range, recognition of the spatial scale at which plants are locally adapted can be notoriously difficult because it requires identification of the major selective agent and spatial scale at which it is acting [18–19]. Locally adapted genotypes can evolve either in response to environmental characteristics that change over short distances, such as soil [18,20–23], or to environmental factors that change over large distances, such as climate [24–26]. To improve our understanding of the relationship between geographic distance and local adaptation, it is required to study adaptive evolution across multiple spatial scales.

Emmer wheat, *Triticum turgidum* L. ssp. *dicoccoides* (Körn.) Thell. is an ideal system to study adaptive evolution across multiple spatial scales. Two distinct features of this species are i) spatially limited gene flow due to predominant self-pollination and localized seed dispersal; and ii) distribution in a wide range of dissimilar ecological niches over both large and small geographic distances. In addition, the species shows high spatial genetic differentiation at various scales [27–32]. The high spatial genetic structuring in emmer wheat can either be the result of spatially limited seed and pollen dispersal or of environment-specific natural selection. The species occupies various habitats in terms of aridity (from less than 300 to more than 1300 mm of annual rainfall), topography (different rockiness, slope steepness) and soil types (basaltic soils on basalt bedrock, terra rossa soil on hard limestone bedrock and rendzina). At the local scale, emmer wheat occupies a spectrum of microhabitats from rocky to flat areas of different slope angle and orientation [33–34]. Habitat differences caused by differential soil moisture, competition and grazing intensity can potentially lead to microscale natural selection and several studies on wild emmer have claimed to find evidence of micro-habitat specialization detectable at the molecular level [30,35–39]. This claim was refuted by Volis et al. (2004) [31] who showed that a relationship between allele frequencies at molecular markers and topography was apparent and solely due to similar scales of topographic variation and seed dispersal. In that study ordination analyses detected a significant effect of spatially structured environmental variation on genetic differences between plants for allozymes, glutenins and quantitative morphological and phenological traits. However, after removal of the spatial component of variation in the analyses, the relationship of the remaining environmental variation with these genetic markers could be explained by chance alone. The detected population genetic structure was in agreement with one expected under isolation by distance as a result of limited gene flow, and topographic autocorrelation was similar to genetic marker autocorrelation, indicating similar scales of environmental heterogeneity and seed flow. On the other hand, no experimental assessment of plant performance has ever been done in emmer wheat, so the question whether local adaptation in this species has occurred and at which scale (micro-habitat or higher scale) has remained unanswered.

Reciprocal-transplant experiments employing cross-relocation of individuals originating from different habitats are the classical approach to test for spatially heterogeneous selection [40–41]. In these experiments higher fitness of native vs. alien genotypes in their respective environments is evidence that populations are locally adapted. However, as was pointed out by Kawecki and Ebert (2004) [7] in their conceptual review of local adaptation, there is no single answer to the question of how fitness should be measured. The most common approach is to use one or more individual traits as estimates of fitness. Although a variety of individual performance traits have been used for this purpose (e.g. flowering probability, germination percentage, plant growth rate), the most commonly used traits are stage-specific or life time survival and fecundity [42–44]. The problem with this approach is that fitness-related traits can trade-off with other traits and be under stabilizing (and not directional) selection (reviewed in [45–46]). As a result, different intermediate trait values may be optimal in different locations [7]. This approach can be especially misleading if the habitat's environmental conditions fluctuate over time and create conditions of constantly changing local optima.

Two other approaches overcome this problem by estimating performance of a genotype or a pool of genotypes over time, rather than individual life history components or life time reproductive success. The first alternative approach is to trace the outcome of competition among genotypes representing different habitats and to measure their contribution to the next generation (e.g. [47–48]). This approach is rarely used because of limited availability of specific genetic markers needed for tracing the genotype contribution to the next generation(s). The second alternative approach is to measure and compare the population growth rates of habitat-specific genotypes or pool of genotypes in a given habitat (e.g. [49]). This approach is usually used for species with little intraspecific competition and rapid growth (such as bacteria and insects), but can be used for plants as well [50].

We designed an experiment allowing usage of all the three approaches for testing local adaptation and compared their efficiency. In addition, we assessed molecular and quantitative trait variation and calculated the *Q*_ST_/*F*_ST_ ratio. There are three widely accepted scenarios of *Q*_ST_ *—F*_ST_ comparisons, (i) no effect of selection (*Q*_ST_ ≈ *F*_ST_); (ii) diversifying selection (*Q*ST > *F*ST); (iii) convergent selection (*Q*ST < *F*ST) [51–52]. As gene flow and natural selection are dependant on the geographic scale, the suitability of *Q*_ST_ *—F*_ST_ comparison for making inferences about selection effect can also depend on the spatial scale [53–55]. Therefore we used both the *Q*_ST_ *—F*_ST_ comparison and a direct test for local adaptation through reciprocal transplanting to test whether the results of the two tests corresponded to each other when the tests are applied at different spatial scales.

Our study of local adaptation included sampling in dissimilar habitats at two spatial scales followed by field introduction, and analysis of molecular (SSR) and quantitative trait variation. Our question was whether there is local adaptation at either large or small spatial scale in emmer wheat revealed by the *Q*_ST_ *—F*_ST_ comparison and reciprocal transplanting. The latter has been done as multi-generation tracing of experimental populations composed of genotypes of different population origin. This experimental design introduces two simultaneously present effects on plant performance: the effect of local environment and the effect of competition with other plants of both local and alien origin. Although simultaneous presence of the two effects does not allow estimating each effect per se, this design simulates a situation where non-local genotypes are introduced as a result of gene flow and compete with the locals. In a case of strong local adaptation, the non-local genotypes are expected to be outperformed by the locals.

## Materials and Methods

Israel Nature Reserves and National Parks Authority permitted usage of its land for the experiments. The study did not involve any endangered or protected species.

### Study species and locations

*T*. *turgidum* var. *dicoccoides* (genome AABB, 2n = 4x = 28) (hereafter *T*. *dicoccoides*), wild emmer wheat, is a predominantly selfing grass and the tetraploid progenitor of most cultivated wheats [56]. It is distributed throughout the western part of the Fertile Crescent, an area of ancient agriculture stretching in an arc from the Nile to the Tigris and Euphrates and comprising Israel, Lebanon, Jordan, Syria, Iraq, southeastern Turkey and western Iran [33,57–58], with a center of distribution in the catchment area of the Upper Jordan Valley in steppe-like herbaceous formations of the *Quercus ithaburensis* open-park forest belt [59].

**Fig 1:**
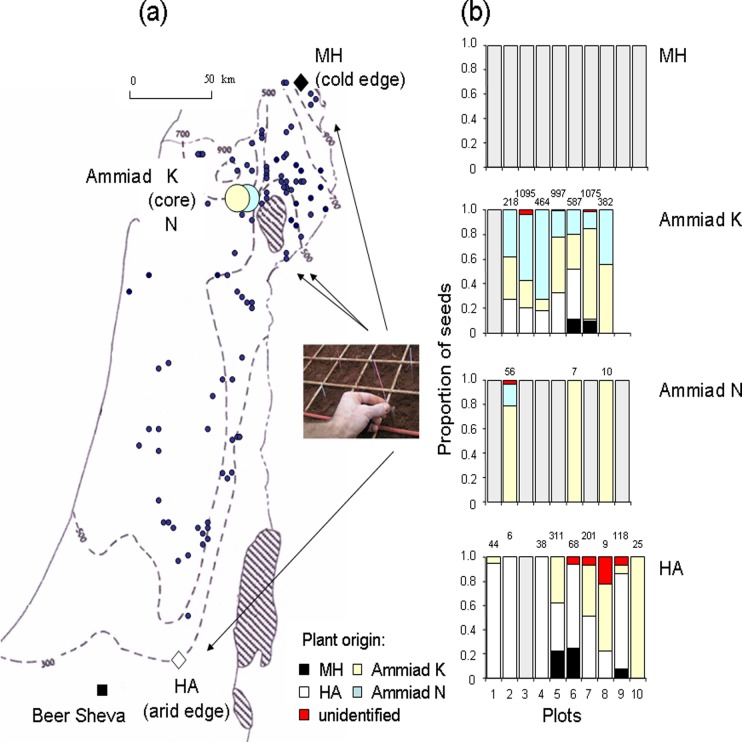
A map of Israel showing isohyets of multiyear averages of annual rainfall amount (mm), distribution of *T*. *diccocoides* and the four study populations (a); and proportions of seeds of different origin four years after planting in each of the four locations in the reciprocal transplant experiment (b). Dots mark known populations of *T*. *diccocoides* based on data of the Institute for Cereal Improvement (Israel). Grey bars denote experimental populations that got extinct. Total number of seeds per experimental population is shown above the bar.

Sampling was done at two spatial scales. A macroscale included two locations with extreme environmental conditions from the two opposite edges of the species distributional range (mountain and semi-desert steppe), and one more favorable distributional core location in the Upper Jordan Valley catchment area (Fig. 1a). The locations were separated by more than 50 km. At Mount Hermon (MH) we sampled the northern-most population in Israel. The site is located on a south facing slope of the mountain at an elevation of 1500 m. The mean annual rainfall is more than 1300 mm (at Majdal Shams, few kms apart). The climate at this location is much cooler than at the other two sampled locations (in contrast to the other two locations the area is covered with snow during winter months).

At Har Amasa (HA) we sampled the southern-most population in Israel on the edge of the Judean desert on an east facing slope at an elevation of about 900 m. The climate is steppical and the mean annual rainfall is assumed to amount to less than 300 mm (383 mm was measured in Daharyyia, about 15 Km northwestwards on Judean Mountains). The plants grow in soil pockets between large rocks.

The third location, the Ammiad conservation site (Ammiad) is north of the Sea of Galilee in Northeastern Galilee at altitudes between 240 and 350 m above sea level [34]. The soil is terra rossa with limestone uneven micro-relief. It features a typical Mediterranean climate with an average annual rainfall of 580 mm (±151 mm SD).

A microscale sampling was performed at the Ammiad location, where we chose two previously identified topographically dissimilar microhabitats [34,72]. Ammiad North (Ammiad N) is located on a moderate north-facing slope at an elevation of 260–280 m with relatively low rock cover (20–60%). Ammiad Karst (Ammiad K) is on a steep south-facing slope of rockier micro-relief (40–80% rock cover) at 320–340 m above sea level. Although these two microhabitats are in close proximity to each other (about 1 km), Nevo et al. (1991) [37] claimed that *T*. *dicoccoides* showed local adaptation detectable at the molecular level.

Sampling of plant material to be used in the experiments was done in 2007. From each sampled plant we took a separately bagged spike and precise GIS coordinates of its location. The number of sampled plants was 70, 68, 57 and 94 (Ammiad K, Ammiad N, MH and HA locations, respectively).

### Reciprocal transplant experiment

In order to test for local adaptation among the three populations separated by more than 50 km from each other (MH, Ammiad and HA) and two microhabitats separated by approximately one km (Ammiad N and Ammiad K), we compared plant differences in fitness using the ‘local vs. foreign’ criterion [7]. Local adaptation was defined as superiority of plants of local origin over foreign plants in their home site. The home site advantage was estimated for both planted individuals and their progeny (i.e. population size over generations). For the latter, the plant origin in the experimental populations was identified with molecular markers.

We screened 27 polymorphic genomic SSR markers [39–30] and selected four SSR markers allowing easy identification of the accession (= genotype) origin to one of the four populations (GWM124, 128, 162 and 169; for details about the markers see [60]). Prior to the experiment, we planted five randomly chosen accessions per population in a greenhouse, to obtain the necessary amount of seeds genetically identical to the mother plant, and remove/reduce maternal effects. The propagated seeds were used for creation of random combinations of accessions to be reciprocally transplanted.

In the transplant experiment, we randomly located replicated plots. Each plot contained 36 seeds arranged at 10 cm intervals in six 50 cm long strips spaced 10 cm apart. In each strip six seeds placed in open from both sides Eppendorf tubes and filled with the soil from the burial site were buried so that tube edge was at ground level. Each plot comprised a set of either 4 accessions representing populations HA, Ammiad K, Ammiad N and MH replicated 9 times, or 3 accessions (populations HA, Ammiad K and MH replicated 12 times) arranged regularly as in a checkerboard. In November 2007, sets of 3 accessions were planted at the MH and HA sites and sets of 4 accessions were planted at the Ammiad K and Ammiad N sites. The vegetation and litter within plots plus 30 cm around was carefully removed. Each tube was tagged with a wire attached to the tube and painted with a fluorescent paint to allow subsequent detection. Ten plots were established at the MH and HA sites and nine plots at the Ammiad K and Ammiad N sites. One plot was subsequently lost at the Ammiad K site due to disturbance. Plant fate was followed throughout the first season and seed germination, plant mortality and number of seeds produced were recorded. The seeds were counted on a plant before shattering, then allowed to fall and further left untouched.

In the fourth season, we estimated each accession's success over time (i.e. how many plants/seeds it produced) using genotyping with SSR markers. For this purpose, after counting the seeds on a plant we collected its fresh leaf for DNA extraction and accession identification.

Home advantage of plants of different origin was tested at each location separately through pair-wise population comparison using aster modeling of individual lifetime fitness [61–62] as implemented in R [63]. Aster models are a significant improvement over previous attempts to model lifetime fitness because they allow (likelihood-based) modeling of multiple components of life history in a single analysis, with an individual's response at each life history stage conditioned upon its response at the previous stage. For the first year assessment, the modeled life-history stages and their statistical distributions were early-season seed germination (Bernoulli), whether a plant reproduced or not (Bernoulli), and total seeds per plant (Poisson). For the fourth year assessment, we had to reshape data for analysis following Stanton-Geddes et al. (2012) [50] because it was not an individual-based but a group-level aster analysis that starts with a 'root' of either 12 (MH and HA locations) or 9 (Ammiad K and Ammiad N locations) as the number of seeds of particular origin sown in the first year. The modeled life-history stages and their statistical distributions were number of plants (Poisson) and number of seeds (zero-truncated negative binomial). As the current aster package automatically accommodates only single-parameter exponential family distributions, the size parameters for the negative binomial distributions were chosen by fitting that distribution (fitdistr function in library MASS, [64] in R) to the conditional distribution of seeds counted. In each aster model comparison Likelihood ratio test compared the fit of the full model to reduced models that sequentially dropped terms.

We calculated origin-specific population growth rate for each plot one year after introduction as λ = (number of seeds produced in 2008) / (number of seeds sown in 2007), and four years after introduction as λ = (number of seeds produced in 2011) / (number of seeds sown in 2007). Because we planted at low density, our population growth estimates approximate finite rate of population increase measured after a colonization event [65]. The 95% confidence intervals for population growth rate per site were calculated by bootstrapping over plots.

### Assessment of quantitative trait variation

Seeds from the plants separated by more than one meter in each of the four populations were used for analysis of extent and structure of genetic variation in 11 quantitative traits. The 54–65 accessions (= genotypes) were sown for each source population and each accession was represented by three individually planted seeds. Because of high selfing rates in *T*. *dicoccoides* [66], progeny of each accession can be considered genetically identical. Seeds were simultaneously germinated at 24°C and transferred into 3 liter pots arranged in a greenhouse at the Bergman Campus, Beer Sheva, using block design. The pots were filled with a commercial potting mixture. During the experiment, the plants received an amount of water equivalent to 1058 mm of annual rainfall. Watering was done twice a week using a drip-irrigation system. The measured quantitative traits included tiller height (TH), flag and penultimate leaf length and width (FLL, PLL, FLW and PLW), spike length (SPL), awn length (AWL), number of spikelets in a spike (NSP), number of days to awning (DAW) and seed maturation (DMT). At senescence, mean spikelet weight (SWT) was obtained from the total number of spikelets and total seed mass per plant.

The structure of variation in phenotypic traits was analyzed after running nested ANOVA by partitioning the total variance into several components. Two random effects included Population/Habitat and Accessions nested within Population/Habitat. The REML procedure was used for calculation of variance components. The *Q*_ST_ estimates were calculated for each measured trait using the equation *Q*_ST_ = V_POP_/(VACC+VPOP) where V_POP_ is the estimated variance component for the population effect and V_ACC_ is the estimated variance component for the accession (= genotype) effect. The average *Q*_ST_ values were provided with 95% confidence intervals obtained by bootstrapping over traits. The variance components were calculated with Statistica 10 and bootstrapping was done with S-plus 2000 software.

### Microsatellite analysis

From the collected samples, only plants separated by more than one meter were used for analysis of extent and structure of genetic variation with SSRs. The number of accessions analyzed for the populations MH, Ammiad K, Ammiad N and HA was 42, 41, 40 and 44, respectively. Genomic DNA extraction followed the modified CTAB protocol of Rogers and Benedich (1985) [67]. Eleven polymorphic nuclear SSRs [60,68] were amplified with polymerase chain reaction (PCR) according to Röder et al. (1995) [68]. The PCR products were detected and sized by the ABI PRISM 3700 DNA Analyzer at the Hebrew University, Jerusalem, Israel. The data were analyzed using Peak Scanner Software v1.0 (Applied Biosystems).

The distribution of genetic variability within and among populations/habitats was investigated by an analysis of molecular variance (AMOVA) using GENALEX version 6.0 [69], and Weir and Cockerham's (1984) *F*-statistics as implemented in GDA [70]. The results were very similar and therefore for multilocus population pairwise *F*_ST_ only the latter are presented. We also estimated population differentiation by the *D* measure [71]. *D* values were averaged over polymorphic loci. The 95% confidence intervals for the population pairwise *F*_ST_ and *D* values were obtained by bootstrapping over loci. *Q*_ST_ was considered to be statistically different from *F*_ST_ when 95% confidence intervals of *Q*_ST_ did not overlap 95% confidence intervals of *F*_ST_.

## Results

### Plant performance in reciprocal transplant experiment

**Fig 2:**
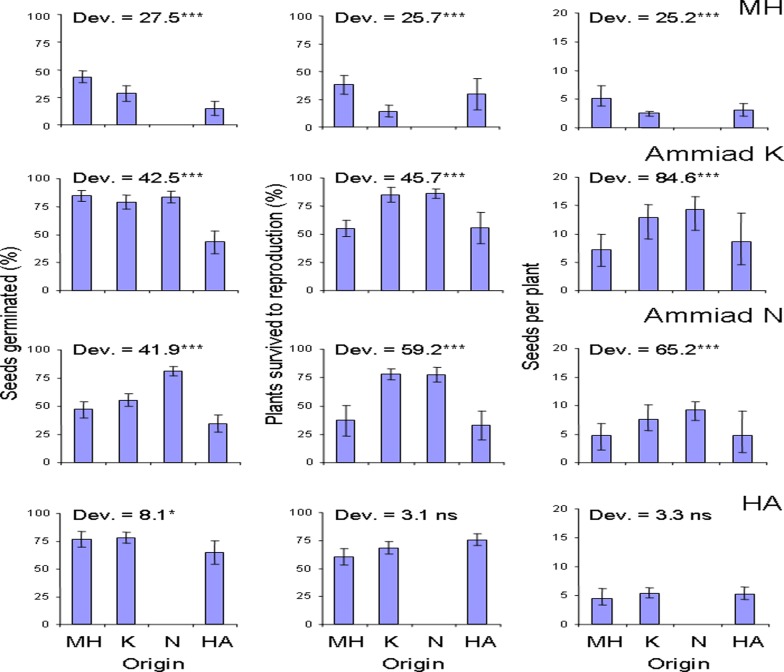
Effect of population origin on seed germination, plant survival and fecundity at the four introduction sites in the year of planting. Bars represent mean values (±SE). Likelihood ratio tests were used to compare the fit of the full model including effects of plant origin and block to the reduced model with block effect only. Test deviance values are provided with the significance level. *** p < 0.001, * p < 0.05, ns not significant.

The effect of population origin on seed germination, plant survival and fecundity at the introduction sites in the year of planting can be seen on Fig. 2. All three fitness components exhibited strong genotype x environment interaction. The HA population had the lowest germination percentage at all four sites. The locals had superior germination percentage at the MH and Ammiad N sites. The Ammiad plants had superior survival till reproduction over the MH and HA plants at both Ammiad locations K and N, but differences in fecundity among the plants of different origin at the two Ammiad locations were less obvious. Analysis of lifetime individual fitness by aster modeling that takes into account all the three fitness components revealed home advantage for the northern edge population (MH) and the core population (both habitats Ammiad K and Ammiad N) when compared with MH and HA populations, while the southern edge population (HA) did not differ in performance from the other two populations (Table 1).

**Table 1:** Summary of results from aster model comparisons testing the effect of population origin on plant performance at each transplant location in the first and forth year after planting.

Model	Model d.f.	Test d.f.	Test deviance	P-value	Model d.f.	Test d.f.	Test deviance	P-value
*First year*					*Fourth year*			
*MH site*								
*MH vs*. *HA*								
Full	3							
Block	12	9	41.5	<0.0001				
Population	13	1	16.1	<0.0001				
*MH vs*. *Ammiad K*								
Full	3							
Block	12	9	28.3	0.001				
Population	13	1	19.5	<0.0001				
*Ammiad K site*								
*Ammiad K vs*. *MH*								
Full	3				2			
Block	10	7	19.2	<0.0001	9	7	49.9	<0.0001
Population	11	1	31.7	<0.0001	10	1	20.2	<0.0001
*Ammiad K vs*. *Ammiad N*								
Full	3				2			
Block	10	7	21.2	0.003	9	7	90.8	<0.0001
Population	11	1	0.4	0.5	10	1	0.5	0.5
*Ammiad K vs*. *HA*								
Full	3				2			
Block	10	7	11.2	0.1	9	7	71.5	<0.0001
Population	11	1	44.2	<0.0001	10	1	6.1	0.01
*Ammiad N site*								
*Ammiad N vs*. *MH*								
Full	3				2			
Block	11	8	19.0	0.01	9	7	4.1	0.7
Population	12	1	48.4	<0.0001	10	1	1.4	0.2
*Ammiad N vs*. *Ammiad K*								
Full	3				2			
Block	11	8	8.1	0.4	9	7	24.5	0.001
Population	12	1	11.0	0.001	10	1	0.6	0.4
*Ammiad N vs*. *HA*								
Full	3				2			
Block	11	8	10.5	0.2	9	7	4.1	0.7
Population	12	1	48.5	<0.0001	10	1	1.4	0.2
*HA site*								
*HA vs*. *MH*								
Full	3				2			
Block	7	4	62.2	<0.0001	11	9	147.2	<0.0001
Population	8	1	1.9	0.2	12	1	21.7	<0.0001
*HA vs*. *Ammiad K*								
Full	3				2			
Block	7	4	29.7	<0.001	11	9	146.6	<0.0001
Population	8	1	0.2	0.6	12	1	4.8	0.03

Notes: Likelihood ratio tests were used to compare the fit of the full model to reduced models that sequentially dropped terms.

Analysis of deviance (-2 log likelihood) and χ^2^ P-values for each model test are listed.

Analysis of group (i.e. population) performance four years after introduction at the large spatial scale detected home advantage for the core population Ammiad habitat K, but not habitat N, and for the southern edge population HA (Table 1). All the experimental populations got extinct four years after introduction at the northern edge location (MH).

At the small spatial scale, within the Ammiad population, in the year of introduction the Ammiad N plants had superior performance over Ammiad K plants in their habitat, while Ammiad K plants had not. Neither habitat was superior to the other habitat in its own environment in group performance four years after introduction (Fig. 1b). Six experimental populations out of nine, and one out of eight got extinct at the Ammiad N and K sites, respectively. At the HA site, one experimental population out of ten got extinct.

In the MH environment seed germination and plant survival until reproduction (but not fecundity) were the lowest among the sites, but the other three introduction sites did not significantly differ in any individual fitness component in the year of introduction (Fig. 2).

**Fig 3:**
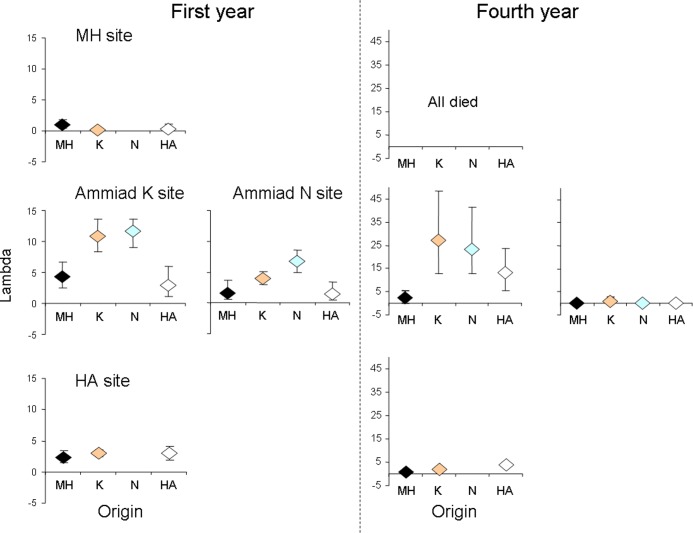
Estimates of growth rate of experimental populations of different origin per introduction site one and four years after introduction.

Comparison of population growth rates showed that the Ammiad population, and specifically its K habitat had the most favorable environment for the study species, and the MH population location had the most extreme one. On the other hand, the population growth rates at the Ammiad N location were even lower than at the species edge HA location (Fig. 3).

### *Q*_ST_—*F*_ST_ comparison

The *H* ^2^ values were above 0.48 for all the traits (Table 2). *Q*_ST_ values were high ranging 0.45–0.91 when the three populations were considered (HA, MH and Ammiad) (Table 2). Subdivision among the two habitats within the Ammiad population was lower, being zero in NSP, SPL, AWL with values above 0.4 only in leaf size traits (Table 2).

**Table 2:** The broad-sense trait heritability (*H* ^2^) and among population and between micro-habitat structuring of genetic variation (*Q*_*ST*_) for 11 quantitative traits.

	TH	DAW	DMT	NSP	SWT	SPL	AWL	FLL	FLW	PLL	PLW
Populations											
*H* ^2^	0.48	0.89	0.87	0.82	0.89	0.71	0.83	0.80	0.80	0.76	0.84
*Q*_*ST*_	0.53	0.61	0.47	0.60	0.91	0.58	0.55	0.45	0.66	0.66	0.67
Habitats											
*H* ^2^	0.49	0.84	0.84	0.59	0.91	0.59	0.76	0.82	0.77	0.68	0.82
*Q*_*ST*_	0.38	0.14	0.18	0.00	0.25	0.00	0.00	0.49	0.70	0.53	0.72

Trait abbreviations: TH tiller height, DAW days to awning, DMT days to maturation, NSP number of spikelet/spike, SWT individual spikelet weight, AWL awn length, FLL flag leaf length, FLW flag leaf width, PLL penultimate leaf length, PLW penultimate leaf width.

Estimates of overall subdivision among the four populations/habitats were very similar for Weir and Cockerham F-statistics and AMOVA (*F*_ST_ = 0.293, CI 0.231–0.368 and *Φ*_ST_ = 0.292, respectively), while Jost's *D* was higher (*D* = 0.391, CI 0.311–0.482). The *F*_ST_ values were consistent among the loci when the three populations (HA, MH and Ammiad) were analyzed, and (with one exception) in analysis of the two habitats (Ammiad K and Ammiad N) (Table 3). Overall, the HA population was most differentiated from the other populations/habitats (pairwise population *F*_ST_ ranged 0.365–0.377) and the differentiation of the MH population from Ammiad K and Ammiad N was about the same as the differentiation between the last two (pairwise population *F*_ST_ 0.192, 0.209 and 0.190, respectively) (Table 4). Differentiation among the three populations and among the two habitats did not differ significantly for either *F*_ST_ or *D* (*F*_*S*T_ = 0.272, CI 0.202–0.355 vs. *F*_ST_ = 0.190, CI 0.113–0.269; *D* = 0.411, CI 0.298–0.513 vs. *D* = 0.242, CI 0.137–0.376).

**Table 3:** Number of alleles, expected heterozygosity (He) and the among population and between micro-habitat structuring of genetic variation (*F*_ST_, *Φ*_ST_ and *D*) for 11 SSR markers.

Genetic parameters	GWM018	GWM186	WMS218	GWM095	GWM251	GWM540	GWM136	GWM537	GWM162	GWM340	GWM124
No. of alleles	5	6	3	5	3	4	3	5	2	2	4
He	0.741	0.794	0.638	0.731	0.523	0.590	0.625	0.667	0.488	0.497	0.712
Populations											
*F*_ST Weir and Cockerham_	0.333	0.100	0.256	0.198	0.330	0.219	0.587	0.165	0.372	0.176	0.248
*Φ*_ST_	0.333	0.100	0.256	0.198	0.330	0.216	0.580	0.165	0.367	0.172	0.248
*D*	0.677	0.273	0.383	0.347	0.346	0.315	0.731	0.344	0.378	0.138	0.474
Habitats												
*F*_ST Weir and Cockerham_	0.085	0.247	-0.009	0.412	0.071	0.082	0.343	0.170	0.175	0.240	0.119
*Φ*_ЗT_	0.085	0.247	-0.009	0.418	0.071	0.088	0.317	0.170	0.166	0.233	0.119
*D*	0.138	0.599	-0.011	0.859	0.037	0.100	0.233	0.265	0.175	0.209	0.277

**Table 4:** Pairwise *Q*_*ST*_ (above diagonal) and *F*_ST_ (by Weir and Cockerham's method) followed by *D* (below diagonal) with 95% CI.

Population/habitat	Population/habitat			
	HA	Ammiad K	Ammiad N	MH
HA	-	0.556 (0.390-0.694)	0.317 (0.220-0.504)	0.713 (0.517-0.832)
Ammiad K	0.369 (0.264-0.470)	-	0.326 (0.174-0.481)	0.797 (0.737-0.867)
	0.512 (0.322-0.684)			
Ammiad N	0.377 (0.205-0.546)	0.190 (0.113-0.269)	-	0.767 (0.690-0.828)
	0.517 (0.258-0.710)	0.242 (0.137-0.376)		
MH	0.365 (0.227-0.492)	0.209 (0.095-0.310)	0.192 (0.099-0.302)	-
	0.508 (0.309-0.690)	0.282 (0.129-0.426)	0.249 (0.108-0.429)	

In both, quantitative trait and molecular (SSR) variation the inter-population component significantly differed from zero in all pairwise comparisons. The *Q*_ST_ was significantly higher than *F*_*ST*_ only in pairwise comparisons of MH with the other three origins (Table 4). In the pairwise comparisons of *Q*_ST_ and *D* only the pairs Ammiad N—MH and Ammiad K—MH differed (Table 4).

**Fig 4:**
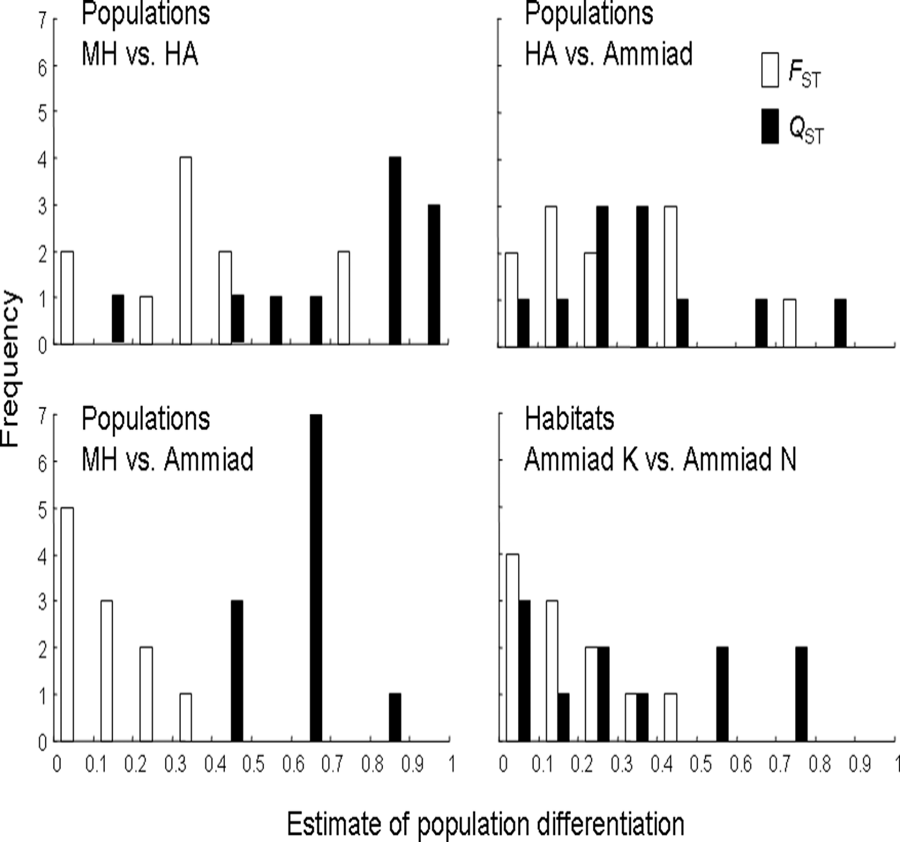
Frequency distribution of locus/trait estimates of population differentiation in pairwise comparisons of the three populations and two habitats.

The among population (i.e. MH, Ammiad and HA) *Q*_ST_ was 0.608 (CI 0.558–0.696) which is significantly higher than corresponding *F*_ST_ = 0.272 (CI 0.202–0.355) and *D* = 0.411 (CI 0.298–0.513). In contrast, the between habitat *Q*_ST_ (i.e. Ammiad N and Ammiad N) did not significantly differ from either *F*_ST_ or *D* (Table 4). The frequency distributions of SSR loci and quantitative trait estimates of regional differentiation for the three population and one habitat pairwise comparisons can be seen in Fig. 4. They show no or low overlap of the two distributions for two out of three pairwise population comparisons and high overlap for the pairwise habitat comparison.

## Discussion

The evolution of local adaptation can be countered, even in highly contrasting environments, by such factors as gene flow swamping locally advantageous alleles, genetic drift limiting selectively important genetic variation and selection regimes favoring a generalist over locally adapted specialists. The relative importance of these factors depends on the geographical scale, as increases in geographic distance between populations are usually associated with increases in environmental differences and genetic isolation. Our findings support this view, as geographic scale was extremely important for local adaptation in *T*. *dicoccoides*.

### Adaptation at small spatial scale

Two habitats within the Ammiad core population, having different topography and vegetation [72] and separated by only 1 km, represented two partly overlapping but distinct gene pools, which is evident in both molecular and quantitative trait variation. On the other hand, the results of tests of individual and group fitness were largely concordant, showing no home advantage for the plants of different origin. These results are consistent with a low intensity of gene flow despite any evidence of differences in local selection between the two habitats. Together, these findings suggest i) no swamping effect of gene flow between the two locations preventing local adaptation at a small spatial scale, and ii) that topography by itself is not the environmental factor that leads to diversifying selection in emmer wheat. Mosaic-type spatial genetic structure is a known feature of *T*. *dicoccoides* in general, and at the Ammiad location in particular [27–30]. Our results agree with an earlier explanation of mosaic-type genetic structure as due to predominant selfing and spatially limited seed dispersal [31,67], and disagree with fine-scale habitat-specific (i.e. topographic) local adaptation in emmer wheat inferred from a relationship between allele frequencies at molecular markers and topography [29–30,37]. As was noticed by Volis et al. (2004) [31], demonstrating an association of allele frequencies with certain environmental variables is not evidence of their causal relationship as a result of natural selection, and can be due to an interrelation of several factors or close overlap of their spatial scales. Findings of Volis et al. (2004) [31] and the present study strongly warn against usage of a correlative approach for any inferences about local selection effect, at least at the small spatial scale.

### Adaptation at large spatial scale

The three populations were genetically and phenotypically distinct. The observed level of population differentiation in SSRs suggests a low to moderate level of historical gene flow between the species core (Ammiad) and margins (HA and MH). However, presently some gene flow appears to exist only between the core and northern periphery. Any gene flow from the core to the HA population is highly unlikely given its extreme isolation (the closest population is about 13 km north).

The transplant experiments revealed that the core population and both edge populations were locally adapted as indicated by a home advantage. Plenty of studies have tested for local adaptation but usually at a single spatial scale (but see [15,18,24,73–74]) with only a few studies assessing population rather than individual performance [50,75–76]. However, detecting local adaptation can be problematic in environments with high spatial and/or temporal heterogeneity, or when local selection is weak. Also, because the life cycle stages are not independent of each other and can be differently affected by the same environment, properly understanding demographic differences between compared populations requires a population approach [75,77]. To this end, tracing population growth rate over several generations and robust analysis of the data through aster modeling is useful. In our study, a home advantage was demonstrated for the two out of three populations (Ammiad and MH) already one year after introduction, but superior performance of the third population (HA) in its own environment was evident only after several generations. At some locations, such as Ammiad N and HA, there was a significant change in home advantage through time, a change that should be attributed to local biotic interactions. The biotic interactions, i.e. competition with other plant species, were absent in the year of planting, but present in the following years. These results highlight the importance of long-term surveys of population growth rate in local adaptation testing even for short-lived organisms.

In general, a reduction in environmental favorability is expected to result in a decrease of survival and reproduction towards the species range edges [78–80]. Indeed, the cold edge population MH had the most extreme environment, reflected by the lowest seed germination, plant survival and population growth rate, and the fact that all the experimental populations at this site got extinct after four years. In contrast, the core population Ammiad, and specifically its Ammiad K habitat had the most favorable environment for the emmer. This was not evident from the individual fitness components, but very clear from the population growth rate. On the other hand, because of spatial heterogeneity, the local conditions within the Ammiad population can be as unfavorable as the conditions at the species edge, and the latter could not be detected without tracing the population fate after several generations. In our study, the arid edge population showed no home advantage in any of the individual performance traits and population size one year after planting, but did show home advantage four years after planting.

In addition, observation of multiple life history stages (e.g. seed germination, plant survival to maturity and seed production) is necessary for understanding the importance of each stage to the populations' dynamics. It seems that seed germination and survival of young plants greatly vary spatially but do not differ much between the core and the arid edge locations, while fecundity in general is reduced at the southern species range. Similarly, local adaptation to the two soil types through differential survivorship and reproductive output was found in several studies [50,81] emphasizing the importance of studying multiple life history components.

Existing ecological models of species distribution explain range limits by an increased probability of extinction and limited availability of suitable habitats (e.g. [82]), decrease in environmental carrying capacity [83–84], or decrease in genetic variance in selectively important traits [7,85]. Our results suggest that all the above features are involved in determining the emmer species range. Both edge populations had reduced variation in quantitative traits important for adaptation [86]. Both range edge populations, and especially the arid edge one occupied only a small area of several hundred square meters of apparently suitable environment, while the core population, despite spatial heterogeneity of its environment, was continuously distributed over an area of several square kilometers. And the marginal environments had either lower carrying capacity (HA) or higher probability of population extinction (MH) than the core environment, although some patches within the distributional core appear to have ecologically marginal conditions. These environmental features could not be identified by a single season study and were discovered due to tracing population size over time.

### *Q*_*ST*_ *-F*_*ST*_ comparison

Population differentiation was high in both molecular markers and phenotypic traits. A high degree of structuring of genetic variation is expected in this species because of its predominant self-fertilization. Self-pollination is a life history trait that limits gene flow and reduces heterozygosity, recombination and effective population size [87–88]. Thereby, it promotes differentiation at all hierarchical levels [89]. Nevertheless, population (but not habitat) *Q*_ST_ exceeded corresponding *F*_ST_ which is in agreement with detected local adaptation of the three populations but not the two habitats. On the other hand, the results of pairwise *Q*_ST_—*F*_ST_ comparisons were consistent with local adaptation of the MH and Ammiad, but not the HA population. This is not surprising, as even reciprocal transplanting failed to detect home advantage of HA plants one year after introduction, and detected local adaptation of this population only after several generations.

### Conclusions

Although for the two out of three populations we detected home advantage already in the year of introduction via measuring life-time plant performance, at the southern site (HA), we detected home advantage only after four years. Our study shows that, although methodologically more challenging, estimation of population growth rate is a better way of testing for local adaptation than estimation of stage- or life time- performance of an individual. Therefore, we advocate the assessing the population growth rate through precise genotyping of individuals with molecular markers and using the powerful statistical approach provided by Aster modeling.
