# Correction: Multi-Approaches Analysis Reveals Local Adaptation in the Emmer Wheat (*Triticum dicoccoides*) at Macro- but not Micro-Geographical Scale

**DOI:** 10.1371/journal.pone.0125258

**Published:** 2015-04-17

**Authors:** The PLOS ONE Staff

There is an error in the third sentence of the sixth paragraph of the Introduction. The correct sentence is: There are three widely accepted scenarios of *Q*_ST_—*F*_ST_ comparisons, (i) no effect of selection (*Q*_ST_ ≈ *F*_ST_); (ii) diversifying selection (*Q*_ST_ > *F*_ST_); (iii) convergent selection (*Q*_ST_ < *F*_ST_) [51–52].

There is an error in the fourth sentence of the second paragraph of the Assessment of quantitative trait variation subsection of the Materials and methods. The correct sentence is: The *Q*_ST_ estimates were calculated for each measured trait using the equation *Q*_ST_ = V_POP_/(V_ACC_+V_POP_) where V_POP_ is the estimated variance component for the population effect and V_ACC_ is the estimated variance component for the accession (= genotype) effect.
